# Climate mitigation policies and the potential pathways to conflict: Outlining a research agenda

**DOI:** 10.1002/wcc.722

**Published:** 2021-06-17

**Authors:** Elisabeth A. Gilmore, Halvard Buhaug

**Affiliations:** ^1^ Department of International Development, Community, and Environment Clark University Worcester Massachusetts USA; ^2^ Peace Research Institute Oslo Oslo Norway

**Keywords:** armed conflict, climate mitigation, sustainable development

## Abstract

Climate policies will need to incentivize transformative societal changes if they are to achieve emission reductions consistent with 1.5°C temperature targets. To contribute to efforts for aligning climate policy with broader societal goals, specifically those related to sustainable development, we identify the effects of climate mitigation policy on aspects of socioeconomic development that are known determinants of conflict and evaluate the plausibility and importance of potential pathways to armed conflict and political violence. Conditional on preexisting societal tensions and socioeconomic vulnerabilities, we isolate effects on economic performance, income and livelihood, food and energy prices, and land tenure as most likely to increase conflict risks. Climate policy designs may be critical to moderate these risks as different designs can promote more favorable societal outcomes such as equity and inclusion. Coupling research with careful monitoring and evaluation of the intermediate societal effects at early stages of policy implementation will be a critical part of learning and moderating potential conflict risks. Importantly, better characterizing the future conflict risks under climate policy allows for a more comprehensive comparison to the conflict risk if mitigation is not implemented and graver climate damages are experienced.

This article is categorized under:

The Carbon Economy and Climate Mitigation > Benefits of Mitigation

## Graphical abstract

Climate policies that emphasize a fair distribution of benefits and compensation for unintended consequences will moderate the risks of violent conflict and future climate impacts.

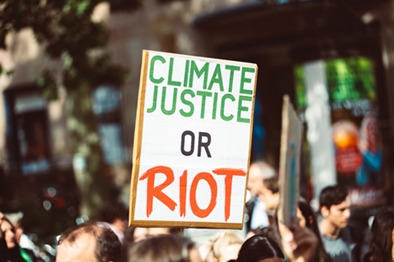

## 1 INTRODUCTION

Over the past decade, a large and increasing number of papers have evaluated possible links between various forms of mainly short‐term climatic changes and different types of conflict and violence (Ide, 2017; Koubi, 2019; von Uexkull & Buhaug, 2021). In efforts to avoid these and other climate‐related risks, the Paris Agreement enshrined the desirability of limiting warming to below 2°C above preindustrial levels, with a goal of a 1.5°C end‐of‐century mean temperature target. Evaluated in the subsequent Intergovernmental Panel on Climate Change (IPCC) Special Report on 1.5°C (SR15), the more stringent 1.5°C end‐of‐century mean temperature target has a meaningful and beneficial impact for the climate system (Rogelj et al., 2018). Presently, it is understood that this target requires net zero emissions by mid‐century. Achieving this target will require substantial interventions that exceed what has already been committed (Brown et al., 2019; Schleussner et al., 2016). To the extent that some of these climate actions may interact with and adversely affect various aspects of human security and well‐being (Campagnolo & Davide, 2019; Jakob & Steckel, 2014; Masson‐Delmotte et al., 2018), there is a critical need to evaluate how patterns of armed conflict may be influenced by climate policy that is consistent with a 1.5°C goal (Buhaug, 2015; Dabelko et al., 2013; Scheffran et al., 2012).

Here, we identify trade‐offs between climate policy and other aspects of development that are known determinants of conflict and evaluate the plausibility and importance of these pathways to armed conflict and political violence. We take the recent SR15 report and associated projections from integrated assessment models (IAMs) as points of departure on the transformations that are required to attain stringent mitigation targets and their interactions with the sustainable development goals (SDGs). We use conflict to encompass outcomes from social unrest to wars within or between states. However, we are primarily concerned about elevated risks of more severe armed conflict as this form of violence has the most devastating economic, social, and political effects with adverse implications for affected societies' ability to respond to climate‐related challenges. Further, we evaluate these risks compared to a counterfactual future where conflict risks are already projected to be lower than today. The climate research community has developed five scenarios known as the shared socioeconomic pathways (SSPs) which outline different plausible future socioeconomic conditions. In our previous work, we showed that the scenario most compatible with climate mitigation—SSP1—also implies much lower levels of armed conflict than today (Hegre et al., 2016). As the scale and scope of the mitigation efforts needed to limit end‐of‐century warming to 1.5°C far exceed both observed policy responses and many analogies—with perhaps the COVID‐19 pandemic introducing conditions that are as sizable as those needed for climate action (Andrijevic et al., 2020)—we also outline a research agenda to better identify the links between climate policy and armed conflict risk. The goal of the envisioned new research portfolio is to provide critical information for policy design to moderate these potential adverse effects, and as such this paper is a first step how climate action interacts directly and indirectly with SDG16—Peace, Justice, and Strong Institutions. This work also informs and interacts with the environmental peacebuilding literature whereby climate mitigation efforts also facilitate discussion and negotiations that may resolve conflict (Dresse et al., 2019; Ide, 2018). We return to these two themes in Section 4 when we outline the research agenda.

## 2 A TYPOLOGY OF CLIMATE POLICIES AND PATHWAYS TO ARMED CONFLICT

Before we delve into security implications of climate mitigation, a brief summary of dominant causes of contemporary conflict is in order. Armed conflict, notably organized uprising by armed nonstate actors against the central state, is usually seen as a reaction to perceived injustice and hardship. Empirical civil war research has identified a range of adverse socioeconomic and political features that are systematically associated with higher conflict risk, such as rampant poverty and poor economic growth, intergroup inequalities, relative deprivation, political discrimination and exclusion, illiberal and corrupt state institutions, weak rule of law, a large population, unstable neighborhood, colonial legacy, and a violent national history (Cederman et al., 2011; Fearon & Laitin, 2003; Hegre & Sambanis, 2016). Climatic changes and shocks may indirectly inform conflict risk by aggravating these conditions, especially in societies dependent on a large climate‐sensitive agricultural sector, although the strength of a detected climate effect to date is modest compared to the influence of main conflict drivers (Koubi, 2019; Mach et al., 2019).

In a similar manner, climate policy tools can be expected to affect the onset, prolongation, and exacerbation of political violence primarily through their influence on societies' economic performance, political stability, and social coherence. However, since conflict propensity is not equally distributed across space, it follows that mitigation‐related impacts represent a security concern primarily for societies already marked by high latent conflict risk, even if these impacts may be products of climate policies implemented primarily in high‐income countries that themselves are unlikely sites of armed conflict. In general, there is more evidence suggesting that climatic hazards affect conflict dynamics (continuation, event frequency, etc.) than conflict outbreak. It is plausible that climate policies will show a similar pattern. Many conflict risks stemming from mitigation are expected to run through the same pathways as climate‐driven conflict risks, such as increasing food prices, poverty, and marginalization. Unfortunately, we are unable to connect specific policies with distinct conflict phases as this would outstrip the literature base. However, we agree that this is important for future theory development and hypothesis testing. Since the purpose of this paper is to motivate a research agenda, we tentatively outline some testable hypotheses that are most supported by literature to guide future theoretical and empirical studies in Section 3.

In Table 1, we present a set of policies and programs employed for mitigation of carbon emissions, the policy outcomes that could promote or dampen conflict, and associated conflict drivers. Our list of climate mitigation efforts, adopted from (Kolstad et al., 2014), and their associated security implications is intended to capture broad, plausible, and representative pathways but it is certainly not exhaustive. This list of policies is derived from a typology of commonly used climate policy tools from the Intergovernmental Panel on Climate Change (IPCC) 5th Assessment Report. To the extent that this report represents the most authoritative synthesis and assessment of the literature, we believe that we have identified the most widely used policy tools. The focus is primarily on domestic policies and the use of international policies, namely offsets and emissions permits, to meet domestic mitigation targets; however, it is also possible to imagine other pathways to conflict risk, including escalating interstate disagreements about cost sharing of mitigation and compensation for loss & damage as outlined in the Paris Agreement (Calliari et al., 2020). We have also restricted our list of policy instruments to those that directly target carbon emissions and do not include other policies and tools with implications for climate mitigation, such as social policy.

**TABLE 1:** Climate mitigation policies with the outcomes that link to conflict pathways and drivers to armed conflict

Policy approaches	Policy outcomes that link to conflict pathways	Conflict driver
Economic instruments—taxes, including a carbon/energy tax (domestic)	Changing the relative price of goods as determined by carbon emissions Altering the viability of economic activities as determined by carbon emissions	Increasing food and fuel prices Loss of targeted livelihoods Adverse distributional effects along preexisting societal cleavages
Economic instruments—International, regional carbon and permit trading, including the Clean Development Mechanisms (CDM) and Reducing Emissions from Deforestation and Degradation (REDD+)	Large financial flows from international trading of permits and emission rights Altering the viability of economic activities as determined by carbon emissions and international demand for carbon permits Altering incentives for the use of land (e.g., REDD+)	Corruption, rent seeking, and displaced economic activity Unequal distribution of benefits locally and internationally Loss of land tenure and displacement, especially of marginalized groups
Economic instruments—Subsidies	Reducing the price of renewable energy and biofuels Eliminating subsidies for fossil fuels	Increased consumer vulnerability to commodity and trade shocks, especially for major food importers Adverse distributional effects
Standards (low carbon fuel standards, sectoral targets on energy, transportation and other energy intensive sectors)	Increasing the price of fossil fuels Increasing the price of specific goods or activities (e.g., electricity or transportation) Altering the viability of specific economic activities Changing agriculture and land use by incentivizing the production of biofuels	Price effects especially challenging for the poor Adverse direct distributional effects by targeted sector Reduced income from international tourism Loss of state revenues for major oil exporters Adverse livelihood effects—loss of small subsistence farmers
Government provision of public goods or services	Altering use of state/national land for mitigation programs Development of infrastructure to support low carbon technologies Support for research, development and deployment related to low carbon technologies	Reduced use of and access to public lands for livelihoods and recreation Changes in patterns of economic growth and employment opportunities with winners and losers

Economic instruments—emission taxes, tradeable emission permits, and subsidies—reduce emissions by increasing the relative price of carbon or energy intensive activities (Aldy & Stavins, 2012; Nordhaus, 2007). Through increasing prices of goods and altering economic patterns, we anticipate increases in conflict risk via pathways that run through economic well‐being. First, the changes in prices as they affect consumers, especially related to food and to a lesser extent other commodities such as energy, have been related to civil conflict and social unrest, although decreases in violence have also been observed due to higher incomes for producers especially in agricultural settings (Fjelde, 2015; Koren & Bagozzi, 2016; Rudolfsen, 2020; Smith, 2014). Over longer periods of time, a clear price signal also supports investments in activities and infrastructure that lead to a longer‐term shift to a lower carbon economy. Such transformation is likely to have substantial effects on employment and livelihoods with distinct winners and losers within and between economies. While the current evidence as often shows net gains in total employment and “green jobs” (Hille & Möbius, 2019; Yamazaki, 2017), the risks of disruption may be especially high to less skilled workers (Marin & Vona, 2019) and more carbon intensive sectors (Carbone et al., 2020), like the fossil fuel industry and other natural resource sectors. The effects of these higher costs and changes to the labor market will also depend on the design of the taxation policy and social policy related to training and employment.

Governments can raise substantial revenues through carbon taxes (or emission trading) which can be recycled for social programs and other lump sum rebates, generating public acceptance for carbon reductions (Baranzini et al., 2017). Acceptance of these taxes may also interact with general levels of public trust (Fairbrother et al., 2019). Taken as whole, however, distributional effects of the tax may have implications for conflict, especially if they imply relative loss of living standards and exacerbate preexisting grievances (Buhaug et al., 2021). For example, rural populations may be more affected than urban residents, as shown in the contemporary and largely nonviolent Yellow Vests movement in France. Additionally, even if taxes target and affect domestic audiences, such effects may have international externalities if they result in behavioral change for a significant number of individuals. For example, if avocados are taxed as an environmental instrument in the European Union (EU), this could hurt poor Mexican farmers.

Economic instruments may also have regional and international security implications. Emission trading is the largest class of environmental trading markets in the world (Newell et al., 2013) with the EU—Emission Trading Scheme (EU‐ETS) being the largest multi‐region system (Ellerman et al., 2016). While an international emission trading scheme as envisioned in the Kyoto Protocol has not emerged, the Paris Agreement laid out a framework for joint mitigation efforts that includes market and trading permits (Aldy et al., 2016). There are also mechanisms that transfer funds to developing countries, such as the Clean Development Mechanisms (CDM) and the Reducing Emissions from Deforestation and Degradation (REDD+) which involve making payments to developing countries to prevent emissions or deforestation that would have otherwise taken place. These programs can generate substantial revenue for the public purse. If these funds are reinvested to promote sustainable and broad‐based programs, such as poverty eradication, education, and public health, then climate policy may be fully consistent with a more peaceful world. However, these funds may also be prone to corruption and patronage, especially at the scale that is projected (Jakob et al., 2015) and may present distributional issues locally and globally (Newell & Bumpus, 2012).

Imposing subsidies on renewables and biofuels or reforming subsidies on fossil fuels can also reduce emissions (Rentschler & Bazilian, 2016). The immediate effect of removing a subsidy is an increased market price of the good. This may have distributional implications by raising consumer expenses relatively more for the poor and separately or concurrently providing opportunities for an elite to capitalize on the new market system when the reforms benefit elite interests (Perry, 2020; Sterner, 2012). Fuel subsidies are often used in autocracies to serve core groups and maintain stability, and the relationship between subsidy cuts, fuel price increase and social unrest is well documented (Natalini et al., 2020). By contrast, the windfalls from removing such subsidies could also be harnessed for development goals (Steckel et al., 2017). One study finds that the estimated cost of ensuring universal access to water, sanitation, and electricity for all people on the planet by 2030 is only a fraction of the accumulated global value of fuel subsidies over this period if current policies are continued (Jakob et al., 2015).

As an alternative to economic instruments, governments may opt for performance standards to reduce future emissions (Burtraw & Woerman, 2013). This regulatory approach is often employed for high carbon electricity generation and transportation sectors as well as for the built environment (e.g., building codes) (Sperling & Eggert, 2014) and may be associated with less public opposition (Rhodes et al., 2017). The effect on consumers depends on the pass‐through of the additional expenses from the producers. The transportation sector, and airlines in particular, may be expected to pass on close to 100% of the additional costs of regulatory compliance to the consumers with follow‐on effects for livelihood and economic performance in countries with high reliance on tourism (Peeters & Eijgelaar, 2014). The still‐ongoing COVID‐19 pandemic has hit the international tourism industry especially hard (Gössling et al., 2020) and may be a forebearer of things to come if future mitigation policies trigger a systematic and lasting shift in leisure travel behavior.

Governments may also enact climate policy through by provisioning public goods or services, specifically the use of national lands for mitigation programs, area‐demanding or intrusive renewable energy production (solar, wind, hydro), afforestation projects, and associated infrastructure (Creutzig et al., 2011). However, use of national parks in particular has been criticized for providing unequal benefits and even direct conflict as the use is shifted to conservation (Carter et al., 2017; Le Billon & Lujala, 2020; Scheba & Rakotonarivo, 2016).

As this paper focuses on the deep decarbonization and net zero emission pathways, we emphasize the conflict potential of mitigation efforts. However, concurrent with these efforts, there will still be the need to adapt to changes that cannot be avoided. The speed and depth of adaptation projects will play a crucial role in minimizing exposure and enhancing resilience in the face of climatic shocks. While it is beyond the scope of this paper to also review the conflict potential of climate adaptation efforts (e.g., Sovacool, 2018), the success of adaptation will play an important role in establishing the future risks for conflict under climate change. A future scenario without adaptation would have a much higher conflict risk, while adaptation conducted concurrently with sustainable development would further reduce these risks (Hegre et al., 2016).

## 3 CLIMATE POLICY, ITS CONSEQUENCES, AND THE POTENTIAL FOR CONFLICT

Here, we discuss several plausible routes from climate policy to conflict embedded in Table 1 above with a specific focus on the mitigation pathways envisaged in SR15. Armed conflict is certainly not an inevitable consequence of interventions to mitigate (or adapt to) climate change; however, the implementation of these actions, when combined with enabling conditions, can increase the risks of conflict. At the same time, armed conflict is also a severe barrier to mitigation and adaptation efforts, increasing climate risks and thereby further raising the benefits of mitigation elsewhere for conflict‐affected societies (Buhaug & von Uexkull, 2021). We evaluate the literature that directly addresses each climate policy and its possible designs to its consequences that might raise concerns for conflict risk through analogies, such as changes in financial flows for oil rents as an analog for transfers under international carbon transfers. To the extent possible, we seek to connect the typology of climate policies with distinct conflict outcomes in the following discussion. However, empirical evidence in support of specific pathways is often limited so these should be regarded as illustrative examples rather than a complete set of plausible outcomes.

Many of the conditions that increase conflict risk, such as lower incomes and weaker and less equitable distribution mechanisms, are more prevalent in the Global South. As a result, many of the theorized pathways from climate policy to conflict are also more likely to dominate in the Global South. Security implications of climate policies are not limited by national boundaries, however. Importantly, mitigation efforts in the Global North—like raising the price of transportation of people and goods affecting tourism, the cost of fuel, and the cost of importing food—can have profound impacts on economies in the Global South. However, such dynamics also may have spillover effects on the Global North and increase tension between perceived winners and losers of international climate policy. Moreover, as the burden of mitigation falls on wealthier states (the major polluters), and mitigation costs result in welfare loss, a relative increase in the baseline risk of social resistance can be expected. While this resistance may result in delays or failures in implementing committed climate policies, middle income countries in transition from autocratic to democratic systems can be at especially high risk of conflict and societal instability and may be particularly affected by restrictive and poorly structured climate policy (Gartzke, 2012).

### 3.1 Effects on economic growth and unequal distribution of economic burdens

In the broadest sense, there is the concern that the mitigation of GHGs will reduce overall economic performance (GDP) by diverting resources from other economic activities, compared to the counterfactual contemporaneous society with less costly mitigation policies. Provided citizens are aware of this cost, this underperformance would subsequently promote conflict risk through any number of mechanisms, including weakening the ability of the state to reduce grievances and reducing the opportunity cost to participate in conflict for individuals and groups by reducing incomes, relative to the counterfactual (Collier, 2004; Fearon & Laitin, 2003). While estimates of the costs of climate policy vary dramatically, the distribution of these costs is unequal between countries as well as within a given country (Boccanfuso et al., 2011; Tavoni & Tol, 2010). As socioeconomic inequality can generate intergroup grievances and social resistance escalating into civil conflict (Cederman et al., 2011; Fjelde & Østby, 2014; Østby, 2008; Scheffran & Cannaday, 2013), climate policies that have distinct differences in impacts across identity groups (e.g., between rural and urban populations or majority vs. minority groups) may be at least as important as the larger macroeconomic effects when assessing the risks of conflict (Markkanen & Anger‐Kraavi, 2019). The actual or perceived impression that these policies will lead to unequal burdens, especially among groups that already are experiencing the effects of inequality and marginalization, may also be a relevant intersection of climate policy, inequality, and populism (e.g., The Yellow Vests movement). Whether anti‐government protest movements result in major armed conflict often depends as much or more on the level of repression by police and security forces as on the nature of the underlying grievances motivating collective action (Ives & Lewis, 2020).

In developed states, relatively lower and more equitable economic growth has also been conceptualized as facilitating mitigation and adaptation efforts. Known as the SSPs, these scenarios outline combinations of population, education, and GDP levels through to 2100 (Dellink et al., 2017; O'Neill et al., 2014). SSPs with more inclusive economic growth where the benefits are shared more equitably result in lower levels of baseline conflict (Hegre et al., 2016). Since one of the most important predictors of conflict is the country's history of conflict, lower baselines risks are of critical importance for understanding future risks. It is more challenging, however, to conceptualize the form of this economic growth over time, and the SSPs may be overly optimistic for current hotspots of conflict and instability (Buhaug & Vestby, 2019). All else equal, lower than expected levels of economic convergence between developed and developing countries will increase the costs of an optimal climate policy today as future generations will have less wealth to offset future damages (Budolfson et al., 2019). Many of the same dynamics that come into play for climate impacts may also affect the poor under climate policy, such as changes in prices and livelihoods (Rao et al., 2017).

Climate policy effects on transportation and tourism may also be a mechanism that reduces overall economic growth in tourism dependent countries and, as this travel is often from North to South, further perpetuate income inequality. Air travel in particular has been identified as an important source of hard‐to‐mitigate emissions due to growth in demand and limited opportunities for fuel substitution (Gössling & Humpe, 2020; Janić, 2018; Schäfer & Waitz, 2014). Of particular concern is the potential that higher ticket fares would have a large, negative effect on tourism (Scott et al., 2015). As tourism can account for a large portion of the economy in many low‐ and middle‐income countries, this unintended consequence may increase economic inequality especially along North–South divides (Peeters & Eijgelaar, 2014). As tourism can also be part of stabilizing post‐conflict settings (Becken & Carmignani, 2016), a reduction in tourism may further increase the overall propensity of conflict in vulnerable settings. In the near‐term, there may also be nonlinear relationships. For example, lower levels of global mitigation efforts combined with an arrival levy that is earmarked for climate adaptation may generate overall gains (Pentelow & Scott, 2011).

### 3.2 Food prices, agriculture, and livelihoods

Renewable energy sources and exploiting the potential of land to sequester carbon, especially by preserving existing forest stocks and afforestation, as well as by coupling biomass with carbon capture and storage to generate energy with net negative CO_2_ emissions, are needed to meet a 1.5°C target (Fuss et al., 2014; Shukla et al., 2019; Smith et al., 2015). These multiple demands for land for mitigation are unlikely to be met with converting agricultural land. Combined with increasing demand for food in response to growing population and shifting diets, these forces may contribute to driving up the market prices for many agricultural commodities (Daioglou et al., 2019). Given the importance of land to climate policy and the potential interactions, the research community has widely investigated the effects on food availability, food prices and agricultural livelihoods, and to a lesser extent on land tenure and resources as it relates to the production of renewable energy (Fuss et al., 2018; Smith et al., 2013).

This interaction has been investigated in some detail in IAMs (Fujimori et al., 2018; Hasegawa et al., 2015; Mouratiadou et al., 2016) and through an inter‐comparison of agro‐economic models, known as AgMIP (Lotze‐Campen et al., 2014). Estimates of the magnitude of the effects in climate policy on prices in global markets range from modest to larger than the effects projected under climate change scenarios with even larger effects on regional and local prices (Fujimori et al., 2019). The effect of higher food prices also interacts with higher incomes for rural, agricultural households. This could offset the local effect of the price increases for farming households; however, urban laborers as well as rural consumers would be adversely affected (Golub et al., 2013).

Although climate change mitigation is often viewed as an important strategy to improve future food security through curbing climate‐related yield loss, mitigation policies also can expose consumers to additional food security threats through land use‐driven increases in food prices. In one study, global hunger by 2050 has been estimated to affect an additional 160 million people under stringent mitigation policy, relative to the no climate policy baseline scenario, although this is before consequences of climate damages are considered (Fujimori et al., 2019). Higher and more volatile food prices, in turn, is often implicated as a factor in social unrest and political violence, especially as a trigger of initial popular mobilization (Hendrix & Brinkman, 2013). Given the inelasticity of demand for food, rapidly increasing prices can have negative impacts of poorer households' disposable wealth and constitute a powerful source of grievance around which collective action can be mobilized. Arezki and Brueckner (2014) found a strong association between higher international food prices and riots and civil conflict in lower income countries. Subsequent investigations using alternative measures of domestic and international food price levels and volatility across spatiotemporal scales have generally verified the robust effect of higher food prices on increasing risk of social conflict, especially in urban centers (Hendrix & Haggard, 2015; Rudolfsen, 2020; Smith, 2014). By contrast, in agricultural economies, higher food prices are associated with increased income, implying that the food price‐conflict link is weak or even negative for rural producers (Buhaug et al., 2015; Fjelde, 2015).

Although food prices in open markets are sensitive to weather fluctuations, nonclimatic economic forces (from domestic food policies and subsidies to international transportation costs and market speculation) have historically been the dominant drivers of shifting price of food commodities. For example, sharp increases in food prices are often exacerbated by food subsidy systems, especially for food importers (Bellemare, 2015; Trego, 2011). There is also some uncertainty about the uniqueness of food as a determinant of social unrest, that so‐called ‘food riots’ are reactions to hunger and food insecurity specifically, as opposed to frustration about increasing living costs and economic hardship more generally (Sneyd et al., 2013). Similarly to food, other basic commodities, like electricity and fuels, are often subsidized, especially in developing countries. In these situations, the subsidy may be applied as a means of shielding consumers from financial market fluctuations but also to maintain order or convey authority through providing services. Removal of subsidies—either due to the inability of the state to continue to support that financial transfer or through market reforms—may generate a violent response through the price mechanism outlaid above (Rudolfsen, 2020), implying that climate policies that have adverse impacts on food consumers should be implemented with care.

### 3.3 Land and water governance

Land and water will play critical roles in climate mitigation for growing biofuels (Erb et al., 2012), maintaining and restoring existing forests and natural ecosystems (Gren & Aklilu, 2016), and water for crops and hydropower (Mouratiadou et al., 2016), as well as for siting solar and wind energy (Petrova, 2013). Given the large range of conflicts that can arise over land, there is reason for concern that existing governance mechanisms may not be adequate to manage the potential conflicts, especially at the scale projected under stringent mitigation policy.

Direct evidence on the interactions of land‐based climate mitigation efforts and conflict comes mostly from REDD+ projects. Property rights and institutions have often been considered as part of the challenge of maintaining the forest stock that are being used in REDD+ projects (Gren & Aklilu, 2016). However, the ability to maintain these stocks may also be achieved through more coercive and violent means which may be more likely to occur as these stocks are now associated with economic rent (Karsenty et al., 2014). While there are some cases of benefits in terms of preserving and enhancing local food security and livelihoods, there are more cases where the local communities have reduced access to the forests (Froese & Schilling, 2019). This results in either direct violence as local communities seek access to the land or indirectly through loss of livelihood and grievances that may reduce the cost of recruitment for rebel groups (Milne et al., 2019). Afforestation and conservation through the preservation of natural parks may also foster conflict with traditional use (Bergius et al., 2020; Cavanagh & Benjaminsen, 2014).

As identified above, bioenergy and afforestation may lead to higher food prices. Higher food prices, in turn, benefit net producers and small landholders by expanding their income. Similarly, there is potential for smaller agricultural producers to further benefit by growing crops for bioenergy in addition to food markets. However, there is evidence that especially in developing countries, agricultural lands are being consolidated by larger multi‐nationals with central government endorsement (Margulis et al., 2013) and that already marginalized groups are likely to be displaced (Creutzig et al., 2013). Renewable energy, such as wind and solar power, also require the allocation of land for siting. While conflicts of a nonviolent nature have been observed in developed countries over these types of developments, there is some evidence that these developments also could spur conflicts in developing countries such as Mexico (Dunlap, 2017), Brazil (Brannstrom et al., 2017), and Kenya (Schilling et al., 2018) as economic benefits may not accrue to those most affected by the development. The intersection of land rights, livelihoods and marginalization could be fertile ground for conflict, especially to the extent that these projects exacerbate existing grievances (Froese & Schilling, 2019). Land‐ and livelihood‐related conflict of the types envisioned here are usually quite confined, pitting small communities against local governments or nonstate actors, or they materialize as disputes between land user groups.

Water resources, governance, and distributional issues are also associated with an increase in biofuel usage and renewable resources, notably hydropower, as there is the potential for trade‐offs between the demands for water across sectors as well as across borders (Zarfl et al., 2014). The same patterns that promote the appropriation of land, especially by multi‐national and foreign entities, are likely to support the consolidation of water rights (Bossio et al., 2012; Woodhouse, 2012). Both small‐scale and large‐scale hydropower projects are associated with shifts in water availability and river flow, notably downstream, which can adversely affect access to land (Islar, 2012), food and agriculture production, and flood management. Such dynamic has been reported in Africa (Annys et al., 2019), Latin America (Riethof, 2016), and Asia (Rasul, 2014). To the extent that water will be diverted both regionally and over international borders and governance structures are insufficient to manage these new activities, these projects further may generate conflict (Petersen‐Perlman et al., 2017). Historically, water‐related conflicts between riparian states have been managed without use of military force, although the potential for conflicts to escalate into major wars is much higher for interstate disputes than for local intercommunal violence (Bernauer & Böhmelt, 2020; Sovacool & Walter, 2018).

### 3.4 Oil revenues, financial transfers, and political stability

Climate policy has the potential for large effects on government revenues through direct interactions with fossil fuel markets as well as through potential financial transfers under the Paris Agreement by way of carbon markets, internationally transferred mitigation outcomes (ITMO), and climate finance. Under stringent mitigation, the usage of fossil fuels is projected to decrease dramatically. Depending on the relative marginal costs of production, these changes in demand will have unequal effects on the exploitation of fuel fossils and oil revenues globally (McGlade & Ekins, 2015). Additionally, climate policy may introduce new revenue streams with the distributional effects highly dependent on the allocation of emission rights. To facilitate climate mitigation, it has been proposed that the savings from reduced fossil fuel consumption at lower prices be shared with those who may see losses in revenue such as fossil fuel exporting countries (Bauer et al., 2013) as well as compensating countries with high adaptation costs and damages (De Cian et al., 2016). While these arrangements are hypothetical right now, regardless of the distribution, however, it is expected that the funds will flow from North to South. These changes in state revenues from countries with high fossil dependency may generate internal issues of political instability through climate policy financial transfers.

Revenues from oil are linked to weak states through diversion of more inclusive economic growth. In the short‐term, the reduction in fossil fuel demand may lead to instability as government revenues will shrink considerably. Additionally, the burden of these reductions may be very unequal, which, in the absence of compensation, could foster grievance. Rapid decarbonization leads to loss of energy and income and thus lower economic growth (or recession) for economies without ample access to renewable energy sources. This scenario poses a particular threat to many major oil‐exporting states where nonoil sectors remain poorly developed (Dutch disease) (Hendrix, 2017). To the extent that some economies are heavily dependent on these revenues, there may be large effects on the ability to maintain social programs, including financial transfers that underpin political stability. The transition out of oil‐based rentier states in countries, such as in the oil‐producing Gulf region, may unleash severe conflict if the economies of the new political systems are unsustainable without the rents. Over the long‐term, shifting from fossil fuels has the potential for greater stability (Hendrix, 2017), especially if policies are pursued to manage the distributional effects of the economic transition (Muttitt & Kartha, 2020). For oil importers, a reduced reliance on oil imports combined with the falling oil prices due to reduced demand may substantially improve their budgets (McCollum et al., 2013) and even more so, if they can replace the oil imports with domestic power production. Similarly, subsidy reform on fossil fuels would also reduce consumption—jointly reducing emissions and reducing the costs to oil importers (Jakob & Hilaire, 2015). However, a shift from fossil fuel usage to renewables may also result in higher energy prices or issues of access that may cut along existing societal fractures, such as less access to renewable energy for the poor (Labordena et al., 2017).

As part of achieving these mitigation policies, emissions in developed countries may be offset at lower costs by purchasing “carbon credits” in developing countries. These financial flows may compensate some of the foregone conventional economic development in developing countries which could have supported stability, especially in transition economies (Gartzke, 2012). These transfers, however, may or may not have the same pacifying effect as other forms of economic growth. First, these funds may have a similar effect to fossil fuel revenues in that they divert other economic activity and amplify incentives for corruption and patronage. Second, they may provide receiving governments with a means of favoring certain groups over others, especially in ethnically fragmented countries where political power and privileges are shaped by ethnic affiliation. In contrast to oil that is primarily purchased by private firms in the market, carbon permits could be sold through rule‐based organizations with binding conditionality for the use of the rents. While the concerns around financial flows from carbon markets remain speculative as these markets have not developed, finance for mitigation and adaptation are increasing. Limited empirical evidence has found that these funds have promoted “green” economic growth with lower emissions (Carfora & Scandurra, 2019). However, in the absence of mechanisms to ensure good governance and equitable allocation of these funds, there is the potential that they may promote conflict (Jakob et al., 2015). As existing structures are often discriminatory, state‐based allocation may promote or, at a minimum, further entrench grievances. Funneling funding directly to local groups has been proposed as a means to address this challenge (Colenbrander et al., 2017).

Finally, while in the long‐term, reducing dependence on fossil fuels will likely have positive geopolitical outcomes, climate policy may also introduce new markets, specifically for rare‐earth elements, which may generate new geopolitical conflicts. New forms of energy generation, specifically the use of batteries for energy storage, may require large amounts of rare earth elements (Deetman et al., 2018). The geographic concentration of some of these elements in a small number of countries has led to concerns about geopolitical supply disruptions (Habib et al., 2016) and changes in the flows of capital (Li et al., 2020), although the conflict potential of these arrangements is less clear (Froese & Schilling, 2019; Paltsev, 2016). Generally, it is expected that the potential for a resource curse is low for most countries with these reserves (Månberger & Johansson, 2019). However, in countries with political instability and weak governance of the mining sector and environment, the extraction of these minerals could be linked to violence, conflict, and human rights abuses (Berman et al., 2017; Church & Crawford, 2020).

### 3.5 Climate intervention: Geoengineering and solar radiation management responses

Unlike the other mitigation pathways that are more likely to occur in a world that is already more peaceful, geoengineering and other climate interventions are a possible climate mitigation strategy that is potentially decoupled from the economic growth or the degree of cooperation in the international community (Victor et al., 2009, 2013). As insufficient action will more likely than not result in a heavy economic and societal toll (Moore & Diaz, 2015), governments may—for any number of reasons—seek to lessen the damages. Geoengineering and solar radiation management encompass a wide range of technologies that focus on interventions in the climate systems to moderate climate change (National Academies of Sciences, Engineering, and Medicine, 2021). One of the most likely strategies—often referred to as solar radiation management (SRM)—is the injection of small reflective particles into the stratosphere to increase the fraction of sunlight reflected back into space to offset the temperature increases from CO_2_—has been given more substantial consideration as it is theoretically sound (Morgan et al., 2013), inexpensive (McClellan et al., 2012), and may be needed to offset emissions even under strong mitigation policies (Moreno‐Cruz & Keith, 2012). These technologies have proven controversial in the policy community as well as with the public (Bellamy et al., 2012).

The security and conflict implications of different climate intervention strategies have been debated within the context of a country or another actor taking unilateral actions that may be perceived as hostile regardless of the intent (Maas & Scheffran, 2012). If climate damages occur earlier than expected, there may be fewer options available to avert these impacts, raising the likelihood of unilateral action (Xu et al., 2018). The effects of these technologies are also very uncertain, and deployment of sulfate particulates to provide temporary cooling may have adverse side effects on other sectors, regions, and actors. Some models find that SRM could reduce inter‐country income inequality by reducing the worse damages for vulnerable countries (Harding et al., 2020). By contrast, public discourse suggests that solar radiation management is perceived as more likely to create a world with an increased probability of geopolitical conflict and even present a challenge to democratic governance (Macnaghten & Szerszynski, 2013). As a result, there are now calls for proposals to address these potential tensions in order to establish principles of international law and governance associated with research and deployment through UN General Assembly or Security Council that could be brought to bear in the case of climate emergencies (National Academies of Sciences, Engineering, and Medicine, 2021).

## 4 DEVELOPING A RESEARCH AGENDA

**FIGURE 1:**
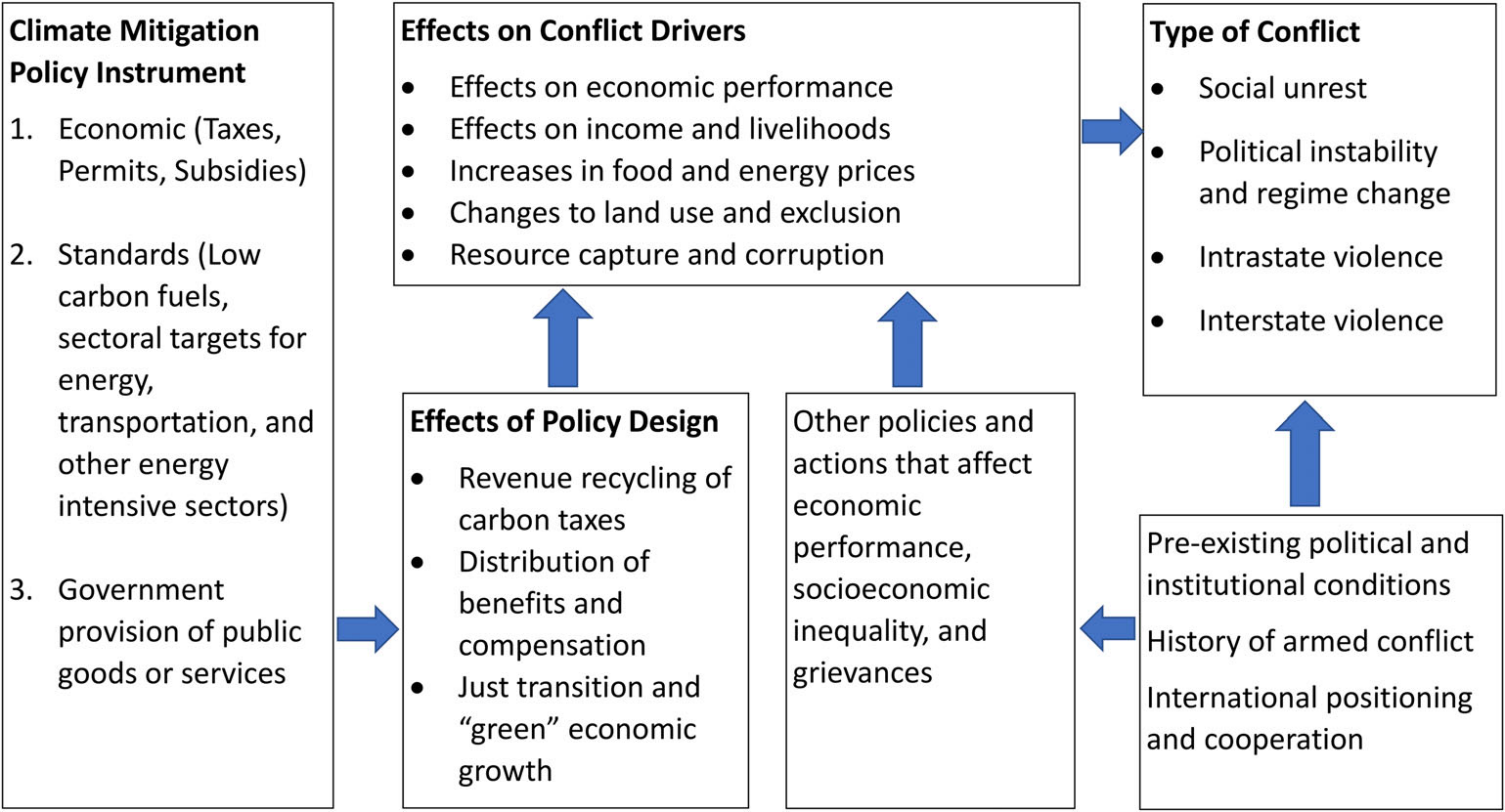
Conceptualizing the links from climate policy to violent conflict

In the discussion above, we illustrate how analogies from existing evidence can provide insights into the effects of proposed climate mitigation policies on conflict. However, there are limits and pitfalls to this approach. Hence, the need for research that can respond more directly to climate policy. In Figure 1, we conceptualize how climate mitigation policy may propagate to increased conflict risks through the selection and design of instruments as well as the interactions with other social and economic policies, preexisting societal structures, and armed conflict history. Our objective with this figure is to extend the pathways in Table 1 to highlight the policy levers that will moderate or amplify conflict risks. An evaluation of the relevant actors and societal structures shaping the relationship between climate policy and insecurity outcome is especially important in the consideration of whether violent conflict is likely, and if so, the type of violence that may result from these new policy environments.

First, we see opportunities for research activities that can provide near‐term information on how climate policy can interact with conflict and its drivers: (1) Investigation of how policy influences perceptions of grievance and attitudes toward violence; (2) Embedding the consideration of conflict risk in the monitoring, verification, and evaluation (MVE) plans for new climate policies and projects, and (3) Identifying and developing natural experiments to look at the effectiveness of climate mitigation interventions.

Research that exploits existing survey data as well as directly asks individuals about their attitudes toward violence has shown promise as far as elucidating how changes in the environment and climate may propagate to violence (Linke et al., 2017). Specifically, this research design offers more opportunities to identify socioeconomic and institutional features that can be more difficult to capture (Rustad, 2015). While surveys of public opinion related to preferences for different climate policies instruments are not new, this type of approach could be adapted to look at the acceptability and effect on attitudes related to violence for different policy tools as well as provide valuable information about moderating policy levers. For example, given the scope of the challenge and the need to reorient many societal activities—potentially through high carbon prices, a redistribution of revenues is likely necessary to moderate the impacts as well as reduce opposition from interest groups (Michaelowa et al., 2018). However, even when revenues are recycled, there has still been opposition, suggesting that a crucial aspect of making these policies desirable has been overlooked from worldviews to employment (Bechtel et al., 2017). To the extent that broad based support for climate policy will reduce the potential for conflict, understanding these perceptions may be especially valuable in guiding efforts related to the “just transition” which appeals to ensuring that climate action is consistent with issues of equity and justice.

We also propose embedding conflict as an outcome of interest at the early stages of implementation of climate projects to develop indicators and participate in the monitoring and evaluation of conflict risks. There are increasing calls to improve the evaluation of the effectiveness of interventions that aim to address climate impacts to reduce conflict (Gilmore et al., 2018; Mach et al., 2020). This also applies to climate policy interventions, especially in locations which are already fragile or have past conflict history. Developing more systematic indicators and monitoring would provide earlier information related to conflict and facilitate more rapid improvement of policy structure. This approach could be especially beneficial for offsets related to land use changes in developing countries or for international efforts where an agreed upon monitoring system could establish responsibility and diffuse disagreements. Further opportunities exist for identifying how these climate mitigation efforts may foster post‐conflict stabilization and attenuate the pernicious effects of armed conflict histories on future conflict risks, especially through generating economic growth and employment (Krampe, 2017). Monitoring the effects of climate policy in conflict and post‐conflict settings could also provide opportunities to refine the theory and practice of environmental peacebuilding (e.g., Ide, 2020). Along these lines, we also encourage natural experiments as climate policy and related interventions are being developed and applied differently across jurisdictions and experienced differently across space. For example, Stokes (2016) identified an opportunity to leverage proximity to a renewable energy project for a natural experiment related to public opposition expressed through subsequent voting against the incumbents. A similar opportunity may exist for climate policies with conflict as an outcome variable such as the conversion of land to national parks for carbon sequestration purposes.

Second, we encourage the development of richer scenarios of the conditions under which we will be enacting these climate policies. The SSPs provide a helpful starting point for analysis of climate impacts; however, they are static such that one cannot easily model feedback effects such as the effect of conflict on economic growth (Hegre et al., 2017). Modeling these feedbacks may be key to providing much needed guidance on where conflict risk may coincide to impede climate action. The SDGs can also be leveraged to generate plausible futures to 2030 and beyond. The SDGs are a multi‐dimensional set of indicators that lay out a vision for a more sustainable and peaceful society. However, there are synergies and trade‐offs across the SDGs with the effects being generated by complex interactions of different mitigation measures and their management (Hegre et al., 2020; Kroll et al., 2019). Elaborating these trade‐offs within the context of SDG16 could further motivate peace by shifting the focus from narratives of more catastrophic futures to those that are more resilient to climate change (Barnett, 2018). This will also provide a deeper understanding of the counterfactual pathways for conflict risks which could support a more rigorous scenario assessment to explore conflict sensitivity not only to warming but also to adaptation and mitigation responses (von Uexkull & Buhaug, 2021).

Finally, we highlight the importance of extending research at the intersection of conflict and effective institutions. New research has indicated that effective and inclusive governments and institutions can help reduce risks related to extreme events (Tennant & Gilmore, 2020). Research that further elucidates the characteristics of effective institutions will support efforts to improve the capacity of governments to develop and fulfill climate policy goals. Effective governments interact with many aspects of climate mitigation. For example, the importance of political stability has been identified for bioenergy production as a prerequisite for fostering investment and agricultural trade (Fuss et al., 2018). However, the ability to enact, enforce, and support climate policy more generally depends on effective governance and institutions.

## 5 CONCLUSIONS

Mitigation actions consistent with 1.5°C temperature targets are needed to forestall major damages to economic growth and other aspects of wellbeing (Moore & Diaz, 2015). As the evidence for the need for urgent climate action continues to mount, it will be critical to undertake fundamental research as soon as possible. In general, climate mitigation foretells a world with reduced climate and conflict risks. However, as we outline here, the type and structure of the policy approaches may either exacerbate or lessen conflict risks. Engaging researchers, practitioners and decision‐makers in the evaluation of early efforts for their conflict potential is critical to aligning climate policy with a more peaceful world.

We are not the only paper to look at trade‐offs along climate policy dimensions (Jakob & Steckel, 2016) and the role of policies in moderating the adverse interactions of climate action with sustainable development goals (Bertram et al., 2018; Mirumachi et al., 2019; Swatuk et al., 2021); however, we are the first to outline how these trade‐offs can influence the propensity for armed conflict systematically across the range of policy instruments that make up the current portfolio for climate mitigation. The pathways from climate policy to conflict described here should not be viewed as exhaustive; nor do they entail inevitable side effects of a policy choice. In characterizing the effect of the policy, we draw on the best available modeling and experience as documented in the literature under the assumption that future conflict will respond similarly to (mitigation‐sensitive) socioeconomic and political risk factors as observed conflict in the contemporary world. However, there are opportunities to address some of these effects in the policy design, such as revenue recycling in carbon taxation approaches to moderate the income and other distributional effects (Beiser‐McGrath & Bernauer, 2019; Swatuk et al., 2021). Understanding how stringent climate policies may interact with conflict dynamics can provide guidance on how to balance more stringent climate policy against other societal objectives and help justify one design over another, especially in contexts that are more vulnerable. It will also serve a critical role in expanding our set of future scenarios by allowing us to better compare the conflict risk if mitigation is not implemented and even graver climate damages are experienced.

## CONFLICT OF INTEREST

The authors have declared no conflicts of interest for this article.

## AUTHOR CONTRIBUTIONS

**Elisabeth Gilmore:** Conceptualization; formal analysis; writing‐original draft; writing‐review & editing. **Halvard Buhaug:** Conceptualization; formal analysis; writing‐original draft; writing‐review & editing.

## RELATED WIREs ARTICLES

Climate change adaptation and peace

Research methods for exploring the links between climate change and conflict

## Data Availability

Data sharing is not applicable to this article as no new data were created or analyzed in this study.
